# Editorial: Audiovestibular disorders in pediatrics

**DOI:** 10.3389/fped.2023.1231803

**Published:** 2023-07-28

**Authors:** Sofia Waissbluth, Jacob Brodsky, Stavros Hatzopoulos

**Affiliations:** ^1^ Department of Otolaryngology, Pontificia Universidad Católica de Chile, Santiago, Chile; ^2^ Department of Otolaryngology and Communication Enhancement, Boston Children’s Hospital, Boston, MA, United States; ^3^ Clinic of Audiology and ENT, University of Ferrara, Ferrara, Italy

**Keywords:** hearing loss, tinnitus, vertigo, pediatric, children, migraine, vestibulopathy

**Editorial on the Research Topic** Audiovestibular Disorders in Pediatrics

Hearing loss is a major global contributor to disability that affects a staggering 466 million people worldwide, with 34 million of those being children. Alarmingly, preventable causes contribute to 60% of childhood hearing loss. The impact of this loss is far-reaching, negatively impacting quality of life and leading to significant educational, social, vocational, and psychological issues. This is especially relevant in education, where hearing loss affects academic performance, school behavior, and learning.

Technology has enabled greater insight into the vestibular system, which affects balance and spatial orientation. Common symptoms of vestibular dysfunction include dizziness and imbalance, which can significantly limit children's participation in various activities and impair their quality of life. Parents may also find such impairments to be alarming and distressing for their children, highlighting the importance of preventing and treating vestibular issues. The Research Topic “Audiovestibular Disorders in Pediatrics” aimed to establish a research forum for audiovestibular disorders in youth, as well as update on latest advancements in audiovestibular assessment, hearing-aid technology, vestibular therapy, and vertigo symptoms among children. A total of five manuscripts are included in this special number. A synopsis of the data is presented in the following paragraphs.

In their scoping review, Gerdsen et al. investigated the impact of cochlear implantation on vestibular function in children by analyzing 14 articles that assessed vestibular function before and after implantation in patients under the age of 18. The authors found that heterogeneity of the data made it difficult to conduct a meta-analysis due to variations in pre- and post-operative testing, the type of vestibular tests used, age of implantation, etiology of hearing loss, surgical techniques, and type of cochlear implants utilized. Of the 14 studies, nine evaluated vestibular function using multiple tests, with the cervical vestibular evoked myogenic potential (cVEMP) being the most commonly utilized (13 out of 14). However, significant variability was observed in the parameters evaluated for cVEMP, including threshold, amplitude, latency, and/or interpeak latency. Ultimately, the authors conclude that vestibular function was either unchanged or showed short-term or permanent decline after cochlear implantation in children. However, the limited and heterogeneous existing literature on the subject precludes any definitive conclusions regarding the overall impact of cochlear implantation on the vestibular system in children. Yong et al. provided a commentary on this scoping review. There is consensus that the impact of cochlear implantation on both objective and subjective vestibular results in children requires further research and remains inadequately comprehended. Therefore, there is a pressing demand for uniformity in perioperative vestibular assessments in children.

Brotto et al., conducted an analysis of vestibular abnormalities and dysfunctions in children diagnosed with inner ear malformations. The researchers identified thirteen relevant studies and categorized their results according to Sennaroglu's classification of inner ear malformations. However, the review of the included articles faced significant limitations due to the restricted sample sizes, heterogeneity of the cohorts, and variable methodologies for assessing vestibular function. Further research is warranted on the relationship between the anterior and posterior parts of the labyrinth, as well as the association between malformations and vestibular symptoms.

Orzan et al., completed a study to assess the impact of newborn hearing screening (NHS) on timing of cochlear implant (CI) surgery of patients with prelingual bilateral profound hearing impairment (BPHI) in Italy. An online questionnaire was used to survey individuals with BPHI born between 1990 and 2018. The results showed that a large majority of respondents had chosen to undergo CI surgery, and most had to move from their region to have the surgery. The study confirmed the effectiveness of the NHS but highlighted issues of late diagnosis and geographical disparities. Social media was a valuable tool for gathering information on health prevention programs.

Teggi et al., retrospectively analyzed data on 95 pediatric patients suffering from episodic vertigo and applied the Bárány Society revised criteria for migraine-related vertigo syndromes in children. They evaluated the phenotypes, bedside examination, and video head impulse tests for three groups: vestibular migraine of childhood (VMC), probable vestibular migraine and recurrent vertigo in childhood (RVC). Applying the revised criteria, 28 patients had VMC, 38 had probable VMC, and 29 had RVC. External vertigo was reported more frequently in VMC patients than probable VMC patients. The duration of vertigo was longer in VMC patients than probable VMC and RVC patients. Cochlear symptoms were reported by some VMC and probable VMC patients. Central positional nystagmus was the most frequent finding during bedside examination in all three groups. Differences in the duration of attacks and accompanying symptoms may suggest different pathophysiological mechanisms.

In summary, the articles presented in this Research Topic provide valuable information regarding audiovestibular disorders in children and highlight the need to improve objective and subjective vestibular testing in various pediatric disorders.

